# 2-Cys peroxiredoxin oxidation in response to hydrogen peroxide and contractile activity in skeletal muscle: A novel insight into exercise-induced redox signalling?

**DOI:** 10.1016/j.freeradbiomed.2020.06.020

**Published:** 2020-11-20

**Authors:** Clare Stretton, Jamie N. Pugh, Brian McDonagh, Anne McArdle, Graeme L. Close, Malcolm J. Jackson

**Affiliations:** a Department of Musculoskeletal Biology, Institute of Ageing and Chronic Disease, University of Liverpool and MRC- Arthritis Research UK Centre for Integrated Research Into Musculoskeletal Ageing (CIMA), UK; b School of Sport and Exercise Sciences, Tom Reilly Building, Byrom Street, Liverpool John Moores University, Liverpool, UK; c Discipline of Physiology, School of Medicine, National University of Ireland Galway, Ireland

**Keywords:** Peroxiredoxin, Muscle aging, Contraction, Hydrogen peroxide

## Abstract

Skeletal muscle generates superoxide during contractions which is rapidly converted to H_2_O_2_. This molecule has been proposed to activate signalling pathways and transcription factors that regulate key adaptive responses to exercise but the concentration of H_2_O_2_ required to oxidise and activate key signalling proteins *in vitro* is much higher than the intracellular concentration in muscle fibers following exercise. We hypothesised that Peroxiredoxins (Prx), which reacts with H_2_O_2_ at the low intracellular concentrations found in muscle, would be rapidly oxidised in contracting muscle and hence potentially transmit oxidising equivalents to downstream signalling proteins as a method for their oxidation and activation. The aim of this study was to characterise the effects of muscle contractile activity on the oxidation of Prx1, 2 and 3 and determine if these were affected by aging. Prx1, 2 and 3 were all rapidly and reversibly oxidised following treatment with low micromolar concentrations of H_2_O_2_ in C2C12 myotubes and also in isolated mature flexor digitalis brevis fibers from adult mice following a protocol of repeated isometric contractions. Significant oxidation of Prx2 was seen within 1 min (i.e. after 12 contractions), whereas significant oxidation was seen after 2 min for Prx1 and 3. In muscle fibers from old mice, Prx2 oxidation was significantly attenuated following contractile activity. Thus we show for the first time that Prx are rapidly and reversibly oxidised in response to contractile activity in skeletal muscle and hypothesise that these proteins act as effectors of muscle redox signalling pathways which are key to adaptations to exercise that are attenuated during aging.

## Highlights

• Hydrogen peroxide is generated by skeletal muscle during contractions.
• Peroxiredoxins (Prx) react with H_2_O_2_ at the physiological levels generated during contractions.
• Prx 1–3 are all oxidised by H_2_O_2_ and contractile activity in muscle fibers.
• Prx2 oxidation during contractions is attenuated in muscle fibers from old mice.
• Prx may act as effectors in activation of redox-regulated adaptations to contractile activity in muscle.

## Graphical abstract

**Figure:**
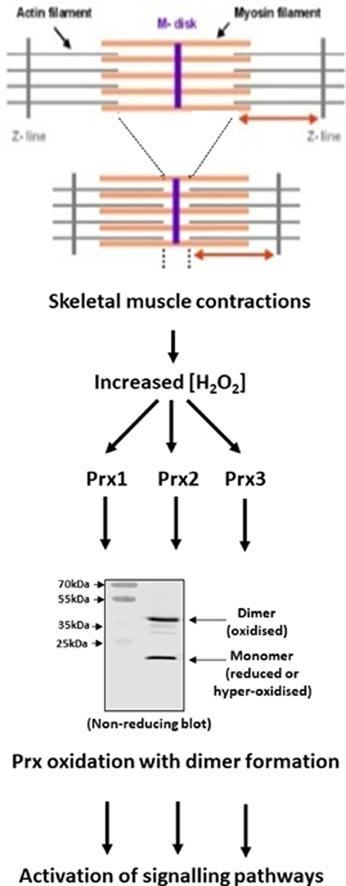

## 1 Introduction

Skeletal muscle adapts to contractile activity through changes in muscle structure, mass and function and several key signalling pathways involved in these adaptations have been identified and characterised [[1], [2], [3]]. Contractile activity of skeletal muscle results in the generation of superoxide and nitric oxide by NADPH oxidase and nitric oxide synthases respectively leading to the formation of secondary reactive oxygen species (ROS), such as hydrogen peroxide (H_2_O_2_), and reactive nitrogen species [[4], [5], [6], [7]]. H_2_O_2_ has been linked to the initiation of the adaptive responses via the activation of a number of signalling pathways and transcription factors leading to the upregulation of genes associated with stress responses, catabolism, glucose uptake and mitochondrial biogenesis [[8], [9], [10], [11], [12], [13], [14]]. However *in vitro* studies linking H_2_O_2_ with signalling protein and transcription factor activation have generally used H_2_O_2_ in a concentration range of 10^−4^-10^−3^ M, which appear to lead to intracellular concentrations of 10^−5^-10^−4^ M [15] and are unlikely to have relevance *in vivo*. Intracellular H_2_O_2_ concentrations are more typically in the range of 10^−9^-10^−8^ M [16] in the quiescent state increasing to around 10^−7^ M in muscle fibers following contractile activity [17,18]. These low intracellular concentrations are therefore not consistent with the concept of H_2_O_2_ generated at the plasma membrane or T-tubules by NADPH oxidase 2 (Nox 2) diffusing through the cell to directly activate redox-sensitive signalling proteins or transcription factors.

One theory that has evolved to explain this discrepancy is that of redox signalling through thiol oxidation initiated by H_2_O_2_. Key components involved in this pathway are peroxiredoxins (Prx) [19]. Prx are present in multiple cellular compartments and are able to scavenge and react with H_2_O_2_ in cells at very low physiological concentrations and are several orders of magnitude more reactive with H_2_O_2_ than many other proteins reported to be redox regulated [20]. Prxs are therefore likely candidates to link contraction-induced H_2_O_2_ generation with activation of redox-regulated signalling proteins and transcription factors. All Prx family members have a conserved cysteine residue, the peroxidatic cysteine (C_P_), in the active site which can be oxidised to sulphenic acid by H_2_O_2_ (shown schematically in Fig. 1). The typical 2-Cys Prx (mammalian Prx1, Prx2, Prx3 and Prx4) also have a conserved second cysteine residue, termed the resolving cysteine (C_R_), which is able to form an intermolecular disulphide bridge with C_P_ resulting in a structural switch to dimer [21]. In solution, the Prx enzymes are found in various oligomeric states [22]. Reduced typical 2-Cys Prxs (Prx1–4) exist predominantly as decamers (5 dimers) or dodecamers (6 dimers). Upon reaction with peroxides, the regions containing the C_P_ and C_R_ become locally unfolded to allow for intermolecular disulphide formation between two subunits with formation of homodimers. This rearrangement destabilizes the decamer/dodecameric state and shifts the equilibrium toward a dimeric state [22]. The intermolecular disulphide can be recycled back to a thiol, thus reducing the Prx by downstream reductants, such as the thioredoxin (Trx)-thioredoxin reductase system.

**Fig. 1:**
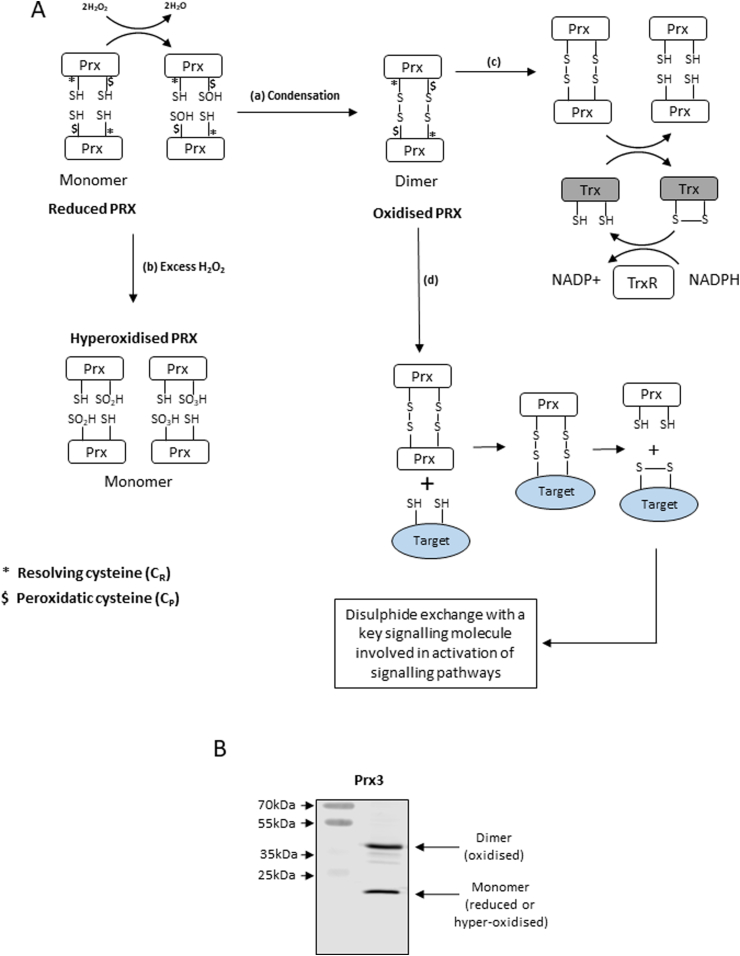
(A) Schematic representation of the oxidation of 2-Cys Prx by H_2_O_2_ and potential oxidation and activation of target proteins by Prx*. H_2_O_2_ oxidises the peroxidatic cysteine free thiol of Prx to sulphenic acid. A condensation reaction occurs between C_P_ and C_R_ of different Prx molecules to form disulphide bridges leading to the formation of Prx homodimers (a). Oxidised Prx in the sulphenic form can also be further oxidised by excess H_2_O_2_ leading to the formation of sulphinic or sulphonic acids, a process termed hyperoxidation (b). Oxidised Prx in the dimeric form can be reduced back to the monomer by thioredoxins (Trx) (indicated in dark grey) which become oxidised and subsequently recycled back to the reduced form by thioredoxin reductases (TrxR) with oxidation of NADPH (c). Oxidised Prx in the dimeric form can alternatively interact with reduced Cys in target proteins (indicated in blue) forming mixed dimers as part of a redox signalling pathway. In this latter process the oxidising equivalent is transferred to the target protein, potentially leading to activation of downstream pathways. The Prx is itself reduced, and reverts to the monomeric form (d). (B) Example of a non-reducing W estern blot used to identify the Prx monomers and dimers showing the 2 forms of Prx3 identified using this technique. *Note, the schematic illustrates oxidation of Prx as observed using non-reducing gels for detection since oligomeric Prx decamers/dodecamers are not seen using this method. (For interpretation of the references to colour in this figure legend, the reader is referred to the Web version of this article.)

A number of redox-activated proteins and transcription factors, such as Signal Transducer and Activator of Transcription 3 (STAT3), Apoptosis Signal-regulating Kinase 1 (ASK1) and Protein Disulphide Isomerase (PDI) have been implicated as potential binding partners for oxidised Prx [[23], [24], [25]]. These proteins are capable of forming heterodimers with Prx before themselves becoming oxidised and passing on the oxidising equivalent to further target proteins through a redox relay culminating in the activation of signalling pathways including other transcription factors (Fig. 1).

The presence of excessive concentrations of H_2_O_2_ can result in the hyperoxidation of Prx C_P_ to sulphinic acid and subsequently sulphonic acid, which is generally considered to be irreversible and non-physiological [[26], [27], [28]]. Hyperoxidation of Prx inhibits the formation of disulphide bridges and results in the inhibition of Prx activity within the cell. The process has generally been considered to be an inactivating step for the protein, but some recent studies have suggested that hyperoxidised Prx can trigger alternate signalling pathways [[29], [30], [31], [32]].

The ability of skeletal muscle from old humans and mice to adapt to contractile activity has been shown to be attenuated compared to that of younger subjects and it has been hypothesised that this may be due to defective redox signalling pathways [33,34]. Our hypothesis was that Prx are key regulators of redox signalling pathways in skeletal muscle during contractile activity and the purpose of this study was to determine the susceptibility of the different Prx isoforms to oxidation during contractile activity and to assess if these pathways were altered by aging. Data obtained indicate that Prx1, Prx2 and Prx3 were oxidised in response to acute treatment of myotubes with low micromolar concentrations of H_2_O_2_ and that in the presence of higher concentrations of H_2_O_2_ the Prx became hyperoxidised. Exposure of intact mature skeletal muscle fibers to a period of repetitive isometric contractions caused a rapid reversible oxidation of Prx 1, 2 and 3 with no evidence for hyperoxidation of any Prx isoforms. Baseline levels of oxidation of Prx2 were significantly lower in quiescent muscle fibers from old compared with adult mice and oxidation of Prx2 in response to contractile activity was also attenuated in isolated mature muscle fibers from old mice. Thus these data provide a novel insight into the potential role of Prx as effectors of H_2_O_2_-induced redox-signalling in response to contractile activity in skeletal muscle and how these processes may become partially attenuated during aging.

## 2 Materials and methods

### 2.1 Cell culture

C2C12 cells were maintained in Dulbecco's Modified Eagle's Medium (high glucose; DMEM) supplemented with 10% FBS, 2 mM l-glutamine, 100 units/ml penicillin and 100 μg/ml streptomycin and grown at 37 °C in 5% CO_2_. Cells were allowed to grow until 80–90% confluent, transferred to medium containing 2% horse serum and then formed myotubes (5–7 days). For hydrogen peroxide (H_2_O_2_) stimulation, myotubes were placed in serum-free medium containing H_2_O_2_ at the concentrations shown in the text and figure legends for the specified times. The myotubes were then washed in PBS and incubated at room temperature for 10 min in PBS containing one of the alkylating agents N-ethylmaleimide (NEM), iodoacetic acid (IAA) or S-methyl methanethiosulfonate (MMTS) at the concentrations specified in the Results section. Solutions containing IAA were adjusted to pH 7.0 with NaOH before use. Myotubes were then lysed in 50 μl RIPA buffer containing protease inhibitors (Roche) and alkylating agent at the specified concentration. Lysates were centrifuged at 12000g for 5 min and the supernatant collected. Protein concentrations were determined using the Bradford assay [35].

### 2.2 Mice

Experiments were performed in accordance with UK Home Office guidelines under the UK Animals (Scientific Procedures) Act 1986 and received ethical approval from the University of Liverpool Animal Welfare and Ethical Review Body (AWERB). Adult (6–8 months) and old (26 months) male C57Bl6 mice were used. Mice were maintained in a temperature-controlled environment and fed a standard laboratory chow diet and subjected to a 12h light-dark cycle. Animals were killed by cervical dislocation and the flexor digitalis brevis (FDB) muscles were removed for isolation of intact single fibers as described below. Tibialis anterior (TA) muscles and gastrocnemius muscles were removed and weighed then immediately cut open to increase the exposed muscle surface area and placed in PBS containing alkylating agents at concentrations specified in the figure legends for 10 min at room temperature. Muscles were then snap-frozen in liquid nitrogen and stored at −80 °C prior to analysis. Frozen muscles were ground under liquid nitrogen and lysed in RIPA buffer (50 mM Tris-HCl PH 7.4, 150 mM NaCl, 0.25% deoxycholic acid, 1% NP-40, 1 mM EDTA and protease inhibitors) containing alkylating agent at the specified concentrations. Lysates were centrifuged at 12000g for 5 min and the supernatant collected. Protein concentrations were determined using the Bradford assay [35].

### 2.3 Isolation of single skeletal muscle fibers

Single intact viable muscle fibers were isolated from flexor digitorum brevis (FDB) muscles based on a method described previously [36]. Muscles were placed in 4 mg/ml type I collagenase in minimum essential medium (MEM) containing 10% FBS, 2 mM l-glutamine, 100 units/ml penicillin and 100 μg/ml streptomycin. Both muscles from each mouse were incubated in the solution for 2–3h and shaken every 30 min to improve digestion of the connective tissue. Intact fibers were separated by centrifugation at 600*g* for 30s. This step was repeated twice and after each centrifugation supernatant was removed and replaced with fresh culture medium. Fibers were plated on to 35 mm dishes pre-coated with ECM Gel from Engelbreth-Holm-Swarm murine sarcoma (Sigma Chemical Co.) and allowed to adhere for 30 min and MEM culture medium was added. Fibers were incubated for 18–24h in culture medium at 37 °C in 5% CO_2_ before use.

### 2.4 Electrical stimulation of contraction of single muscle fibers

Isolated FDB fibers were placed in serum-free medium and were stimulated to contract using field stimulation as described previously [5]. Briefly, platinum electrodes were placed in the culture dish and provided trains of biphasic square wave pulses of 2 ms duration for 0.5s repeated every 5s at 50Hz and 30V for time periods specified in the text and figure legends. Fibers were either harvested immediately after contractions for Western blot analysis or maintained in culture in a quiescent state at 37 °C for specified times before harvesting, at which time fibers were treated with alkylating agents and lysed as described for C2C12 cells above.

### 2.5 Western blot analyses

Forty microgram of lysate for C2C12 myotubes and whole muscles, or 10 μg lysate for isolated muscle fibers was separated by either reducing or non-reducing SDS-PAGE on a 15% polyacrylamide gel. Proteins were transferred to PVDF-F membrane and blocked for 1h in 1% fish skin gelatin (FSG) diluted in tris-buffered saline (TBS). Membranes were incubated with the required primary antibody diluted 1:1000 in 1% FSG containing 0.1% Tween-20 overnight at 4 °C and then IRDye® 800CW goat anti-rabbit IgG (H + L) secondary antibody (Licor Biosciences) diluted 1:10,000 in 1% FSG containing 0.1% Tween-20 for 1h at room temperature. Imaging was carried out using the Odyssey CLx Imaging System (Licor Biosciences) and densitometry analysis carried out using Image J (NIH). Antibodies used were Prx1 (Abcam ab41906), Prx2 (Abcam ab109367), Prx3 Abcam ab73349), Prx4 (Abcam ab184167 and ab59542), Prx3-SO3 (Abcam ab16830) and GAPDH (Sigma G9545).

### 2.6 Statistical analysis

A one-way ANOVA was employed to analyse differences between Prx dimerization (observed in non-reducing SDS-PAGE conditions) across time for H_2_O_2_-treated C2C12 myotubes and FDB fiber stimulation. For comparisons between FDB fibers with stimulation from adult and old mice, a two-way ANOVA was utilised to examine the effects of age and time. If any significant F values were observed, least-significant difference (LSD) tests were performed to determine where any significant differences occurred. An α value of P < 0.05 was used for all tests. All statistical analysis was performed with Prism (v 8.2.1, GraphPad, San Diego, CA, USA) and SPSS (v24, IBM, Armonk, NY, USA). Data are presented as mean ± SEM.

## 3 Results

### 3.1 Alkylation of cells prior to lysis

Prior to commencing the H_2_O_2_ or electrical stimulation experiments, it was necessary to optimise the alkylating agent used to prevent artefactual oxidation of Prx during lysis and sample preparation in both cultured muscle cells and muscle. Three different alkylating agents were investigated. C2C12 myotubes and gastrocnemius muscle were pre-treated with PBS containing increasing concentrations of NEM, IAA or MMTS as indicated in Supplementary Figs. 1 and 2 before being lysed in the presence of 100 mM NEM, 20 mM IAA or 100 mM MMTS. The relative levels of reduced and oxidised Prxs were then assessed by non-reducing western blotting. It is important to note that the native oligomeric reduced Prx decamers or dodecamers are not seen on non-reducing gels and the reduced Prx are seen as a monomer. Two prominent bands were therefore observed for each Prx, a lower band at 20–25 kDa consistent with Prx monomers (the reduced form of Prx, marked as M on the Figures) and an upper band at 35–50 kDa consistent with Prx dimers (the reversibly oxidised form of Prx, marked as D on the Figures). Results are presented in the bar graphs as the percentage of the total Prx signal found in the dimeric form. Bands consistent with Prx dimers were observed for all Prx assessed in cell and muscle lysates that were not pre-incubated with an alkylating agent, suggesting that significant levels of oxidation were occurring during sample preparation. Pre-incubation with both NEM and MMTS dramatically reduced the level of oxidation of Prx1, Prx2 and Prx3 in a concentration-dependent manner, with MMTS being more effective than NEM in all cases (Supplementary Figs. 1 and 2).

Prevention of oxidation was observed to be less robust for Prx3 than it was for Prx1 and Prx2. In all cases the apparent Prx4 oxidation remained unaffected by treatment with any of the alkylating agents tested (data not shown). IAA at all concentrations tested was ineffective at preventing Prx oxidation during sample preparation from both cultured cells and muscle tissue. All further cells, fibers and muscles used were therefore pre-incubated with and lysed in the presence of 75 mM MMTS for all experiments.

### 3.2 Effects of H_2_O_2_ on Prx oxidation in C2C12 myotubes

In order to determine the sensitivity of Prx to oxidation by H_2_O_2_, C2C12 myotubes in culture were treated with increasing concentrations of H_2_O_2_ for increasing periods of time and oxidation of the 2-Cys Prx assessed by western blotting (Fig. 2).

**Fig. 2:**
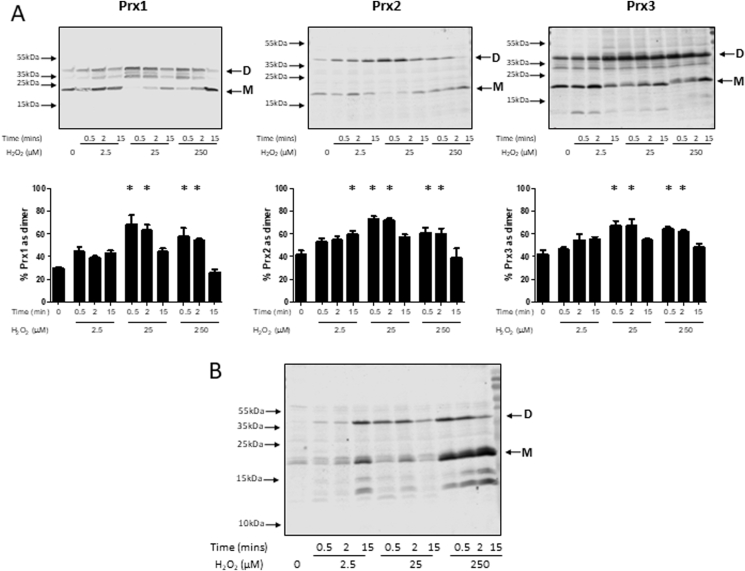
Effects of hydrogen peroxide on Prx oxidation in C2C12 myotubes. C2C12 myotubes were incubated with the increasing concentrations of H_2_O_2_ for defined times and then incubated in 75 mM MMTS for 10 min and lysed in the presence of MMTS. Lysates were separated using non-reducing SDS-PAGE, transferred to PVDF membrane and immunoblotted using antibodies to Prx1, Prx2 or Prx3 (A) or a Prx-SO3 antibody (B). Bands representing the monomers (M) and dimers (D) are indicated. Densitometry analysis shows means ± SEM (n > 3). Results in the bar graphs are presented as the percentage of the total Prx signal found in the dimerised form. *P < 0.05 compared with non-treated cells.

**Fig. 3:**
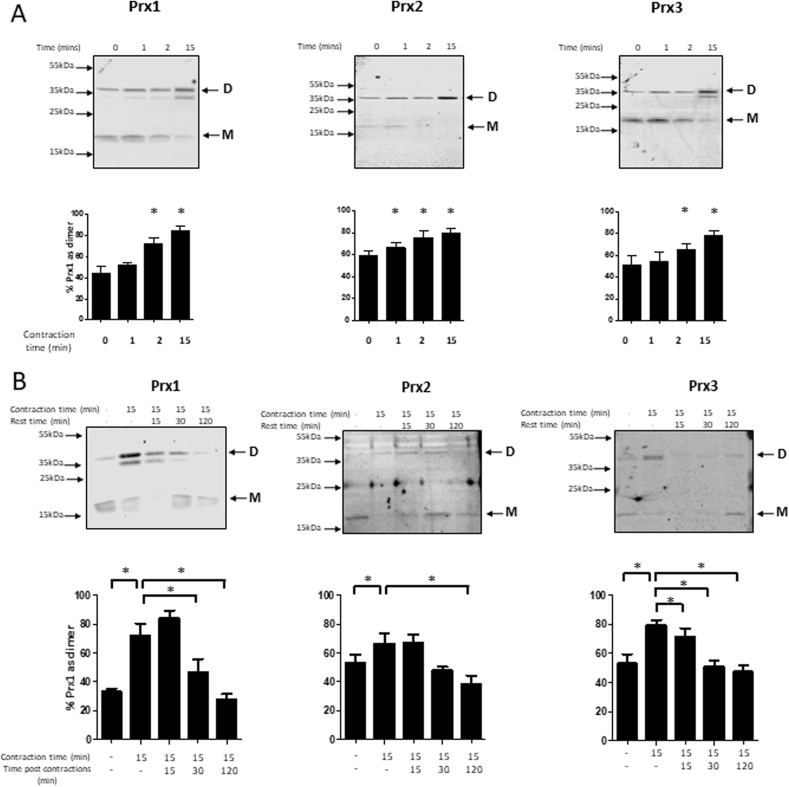
Isolated fibers from the FDBs of adult (6–8 months) male WT mice were subjected to an electrical stimulation protocol as described in the methods for up to 15 min. Fibers were either processed immediately (A) or allowed to rest for the extended periods of time (B) before being incubated in 75 mM MMTS for 10 min and then lysed in the presence of MMTS. Lysates were subjected to Western blot analysis using non-reducing gels and the indicated antibodies. Bands representing the monomers (M) and dimers (D) are indicated. Results in the bar graphs are presented as the percentage of the total Prx signal found in the dimerised form. Graphs show mean ± SEM for at least 3 replicates. In Fig. 3A *P < 0.05 compared with values at baseline for the same Prx. In Fig. 3B *P < 0.05 for the comparisons indicated.

Treatment of myotubes with 2.5 μM H_2_O_2_ resulted in an incremental trend to increased oxidation of Prx1, 2 and 3 determined by the formation of dimers on the non-reducing blots over time, but this only reached statistical significance for Prx2 after 15 min incubation. Following exposure to both 25 μM and 250 μM H_2_O_2_ there was a rapid (within 0.5 min) shift from the monomeric form of Prx1, 2 and 3 to the dimeric form indicating that oxidation had occurred, with some reversion back to the monomeric form occurring after 15 min incubation (Fig. 2A). The non-reducing western blots in Fig. 2, Fig. 3 also showed other bands below the main dimer band that were not non-specific bands. These bands were not included in the quantification, but may represent the single disulphide dimer, or potentially a Prx-thioredoxin dimer, although these possibilities were not investigated further.

Analysis of the western blots with an antibody targeting hyperoxidised Prx showed that some hyperoxidation of the Prxs occurred after 15 min at concentrations of H_2_O_2_ as low as 2.5 μM (Fig. 2B). Incubation with higher concentrations of H_2_O_2_ resulted in an apparent increase in the amounts of hyperoxidation of both the monomer and dimer forms of Prx seen on the non-denaturing blots. The apparent increase in the monomer forms of Prx following treatment with higher concentrations of H_2_O_2_ (Fig. 2A) and the finding that these are heavily hyperoxidised is consistent with previous data indicating that fully hyperoxidised Prx does not readily form disulphide bridges and therefore dimers on the non-reducing blots [37,38].

An assessment of Prx4 oxidation was attempted using this approach, but we were unable to reliably identify Prx4 monomers and dimers despite use of 2 different antibodies. We speculate that this may be related to the very low levels of abundance of this protein in skeletal muscle and lack of sensitivity of available antibodies compared with other isoforms, with Prx4 previously being reported to ~50 fold less abundant that Prx1 and 3 using mass spectrometry approaches [39].

### 3.3 Effects of contractions of Prx oxidation in isolated muscle fibers

Mature muscle fibers isolated from the FDB of mice were subjected to the contraction protocol for increasing periods of time before being lysed and oxidation of the Prx isoforms analysed by western blotting. Both monomeric and dimeric forms were readily detectable for Prx1, Prx2 and Prx3 by non-reducing immunoblotting (Fig. 3). A proportion of all three Prx was observed in the oxidised form in fibers at baseline (i.e. prior to stimulation). Stimulation of the muscle fibers to contract resulted in a rapid increase in the oxidised forms of all three Prx accompanied by a decrease in the reduced forms. In the case of Prx2 the shift to the oxidised form of the protein was found to be significant following 1 min of contractions. The oxidation of all three Prx was significantly increased at 2 and 15 min of contractions (Fig. 3A). No hyperoxidation of Prx isoforms was detected at any of the time points tested (data not shown).

The ability of muscle fibers to reverse the contraction-induced Prx oxidation was assessed by examination of fibers that were quiescent (i.e. not contracted) for up to 120 min following the 15 min contractions. Thus, muscle fibers were subjected to the 15 min contraction period and then maintained in a quiescent state for 15, 30 or 120 min at 37 °C. There was a significant reduction in Prx3 oxidation by 15 min post-contractions, Prx1 oxidation by 30 min post-contractions and all of the three Prx had returned to baseline oxidation levels within 120 min (Fig. 3B).

### 3.4 Effect of age on contraction-induced Prx oxidation

**Fig. 4:**
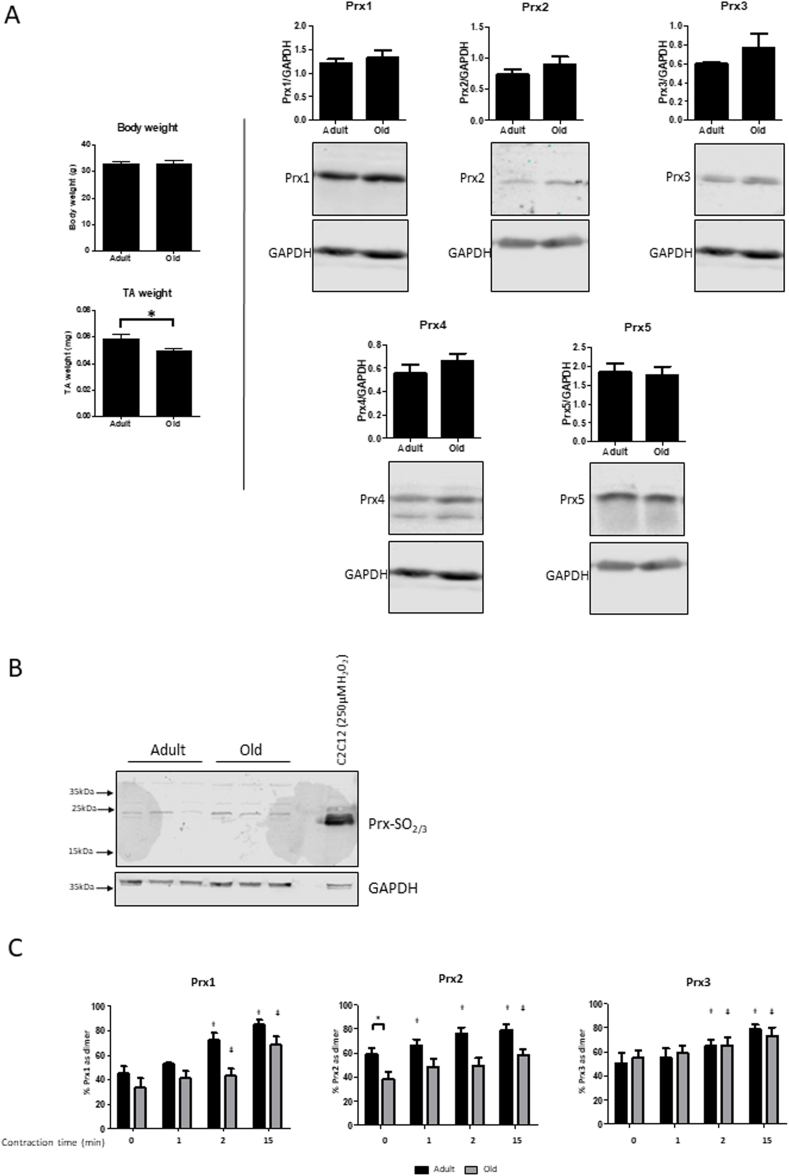
(A) Body weight and TA mass of adult (6–8 months) and old (26 months) male WT mice, *P < 0.05. Example western blots for the Prx1-5 content of the TA muscles from the adult and old mice. Graphs show the mean ± SEM (n = 6 for adult, n = 10 for aged). (B) Western blots for hyperoxidised Prx (Prx-SO_2/3_) contents of the TA muscles from adult and old mice. Lysate generated from C2C12 myotubes treated with 250 μM H_2_O_2_ for 5 min was used as a positive hyperoxidised Prx control. (C) Isolated fibers from the FDB of adult and aged mice were subjected to electrical stimulation for the times indicated before being incubated in 75 mM MMTS and lysed in the presence of 75mmM MMTS. Lysates were subjected to Western blot analysis and antibodies to Prx1, 2 and 3. Results in the bar graphs are presented as the percentage of the total Prx signal found in the dimerised form. Graphs show the mean ± SEM for at least 3 replicates. *P < 0.05, †P < 0.05 compared to adult baseline values, ƚ P < 0.05 compared to old baseline values.

The effects of aging on Prx content and oxidation in skeletal muscle were characterised. Body weight of the mice was unaffected by aging, but the Tibialis Anterior (TA) mass of the old mice was significantly decreased compared with adult mice (Fig. 4A). TA muscles were isolated from adult (6–8 months) and old (26 month) WT mice and total Prx1, Prx2, Prx3, Prx4 and Prx 5 contents were analysed (Fig. 4A). There were no significant differences in the content of any of the Prx isoforms between the TA muscles from adult and old mice. The content of hyperoxidised Prx was also examined in the TA muscles of old and adult WT mice and no differences were seen (Fig. 4B).

Isolated mature fibers from the FDB of adult and old mice were subjected to the contraction protocol above and oxidation levels measured for Prx1, 2 and 3 (Fig. 4C) on non-reducing blots. The baseline level of oxidation of Prx2 was significantly lower in muscle fibers from old compared with those from adult mice. Oxidation of the protein was also much slower in muscles from old compared with adult mice, such that the proportion of Prx2 in the oxidised form remained lower than the adult baseline mean value at all time points. In fibers from old mice the Prx2 oxidation showed a significant increase above baseline values at 15 min post-contractions compared with 1 min in fibers from adult mice. Prx1 and Prx3 oxidation in fibers from old mice showed the same pattern of response to contractions as seen in fibers from adult mice with the increased oxidation reaching significance at 2 and 15 min post-contractions in both cases (Fig. 4C). Levels of oxidation of Prx1 in fibers from old mice appeared lower than those from adult mice at all time points, but this did not reach statistical significance.

## 4 Discussion

Although H_2_O_2_ is potentially damaging to cells, causing oxidative damage to lipids, proteins and DNA, it is increasingly clear that H_2_O_2_ also mediates positive effects in cells such as the activation of adaptive signalling pathways and transcription factors [[40], [41], [42], [43]]. The mechanisms by which this occurs in skeletal muscle remains unclear since the concentration of H_2_O_2_ reported to be required for direct activation of redox-sensitive transcription factor and signalling molecules appears far in excess of what occurs in cells *in vivo* [17,44]. The data presented here indicate that Prx1, 2 and 3 were all rapidly and reversibly oxidised following treatment with low micromolar concentrations of H_2_O_2_ and also in response to a protocol of repeated isometric contractions in isolated mature FDB fibers from adult mice. In muscle fibers from old mice the oxidation of Prx2 was significantly attenuated following contractile activity. Thus, these novel data are compatible with Prx being directly oxidised in response to contractile activity in skeletal muscle and we hypothesise that these proteins act as effectors of muscle redox signalling pathways which are key to adaptations to exercise and partially disrupted during aging.

We initially examined the effects of H_2_O_2_ on members of the typical 2-Cys Prx group of proteins in C2C12 muscle cells in order to confirm that we could detect Prx oxidation following exposure to low concentrations of H_2_O_2_ and determine the relationship of reversible Prx oxidation (detected by dimerization on non-reducing blots) to Prx hyperoxidation. The data obtained indicate that Prx2 could be oxidised by exposure of myotubes to 2.5 μM H_2_O_2_ and Prx 1 and 3 by exposure to 25 μM H_2_O_2_. Hyperoxidation was observed predominantly in the monomeric form but some hyperoxidation was also seen in the dimeric forms of the protein on the non-reducing blots (Fig. 2B). The Prx-SO3 antibody used detects hyperoxidised Prx1-4 and does not differentiate between the different isoforms of Prx and we did not conduct experiments to identify the individual isoforms as part of this study [45].

Prx were rapidly oxidised during contractile activity with the fiber content of Prx2 significantly oxidised within 1 min of commencing contractions. Thus, since each contraction cycle lasts 5 s in the stimulation protocol used, the oxidation of Prx2 had occurred within 12 contractions. The Prx1 and Prx3 isoforms were less rapidly oxidised, but nevertheless this occurred within 2 min of contractions (24 contractions). The contraction protocol has previously been shown to lead to an increase in superoxide generation within 15 min [5,46] with subsequent activation of adaptive responses [43,47]. Previous data also indicate that the source of the intracellular ROS generated by this protocol is membrane-bound NADPH oxidase 2 [46] and the current data therefore imply that following initiation of contractile activity, the NADPH oxidase 2 must be activated to generate superoxide which is converted to H_2_O_2_ within 1 min. Data also indicate that the oxidised forms of Prx were rapidly reduced following the cessation of contractions which indicates Prx oxidation is part of a tightly controlled mechanism to maintain cellular redox homeostasis and regulate intracellular H_2_O_2_ levels. We speculate that this may involve reduction by thioredoxins (Trx), although other target proteins to which Prx transfer their oxidising equivalents have also been identified [21,23,24,48,49].

Data in Fig. 3 indicate that oxidation of the mitochondrial isoform (Prx3) occurred with contractile activity in addition to the cytosolic-located isoforms (Prx1 and 2). Recent studies have implicated NADPH oxidase 2 as the major source of contraction-induced ROS generation [46,50,51], but older [6] and some longer duration studies [51], suggested mitochondria might also be an important source of ROS during muscle contractions. While the source of the ROS that induced oxidation of Prx3 in this model is unclear, the data in Fig. 3 indicate that mitochondria may also play a role in contraction-induced redox signalling.

Prxs have been reported to be the most abundant of the various proteins that can reduce H_2_O_2_ in skeletal muscle. Murgia et al. [39] used a global label free mass spectrometry approach to examine the proteome of single muscle fibers including the relative levels of the glutathione peroxidases (GPx), catalase and thioredoxins (Trx) in addition to the Prxs. In brief, they reported that Prx 1, 2, 3, 5 and 6 show the greatest abundance, being approximately 100 fold greater than GPxs, 20 fold higher than catalase and 5 fold higher than Trx1. Thus in addition to their increased reactivity with H_2_O_2_, the relative intracellular concentrations of Prxs will ensure that these proteins are more likely to interact with newly generated H_2_O_2_ than other scavenging enzymes. We have also previously examined the possible oxidation of Trx and glutathione in skeletal muscle following contractile activity and whilst we found no effect on Trx1 or Trx 2 oxidation, the muscle content of reduced glutathione was decreased following contractions [33].

Murgia et al. [39] also reported on the relative abundance of the different Prx isoforms in fast muscle fibers (which are the vast majority of the fibers found in mouse TA and FDB muscles) and found Prx2 levels were approximately 50% higher than Prx1 and 3. In contrast Prx4 levels were found to be approximately 50 fold lower that Prx 1 and 3. This latter observation may partly explain why oxidation of Prx4 could not be detected in the current study using Western blot approaches.

The method of analysis used in the current study in which total cell lysates were immunoblotted for Prx 1, 2 or 3 did not allow detection of additional bands potentially representing mixed Prx dimers as has been observed in a previous study using a kinetic trapping approach [49]. Identification of target proteins which can form mixed disulphides with oxidised Prx is clearly key to understanding the role of Prx as effectors of muscle redox signalling. Some progress has been made in identifying direct binding partners of the different Prx isoforms, but the mechanisms by which Prx are able to trigger signalling pathways leading to transcription factor activation remain unclear. The transcription factor STAT3 has previously been shown to interact directly with Prx2 in HEK293T cells [49] suggesting that, in some cases, transcription factors may be activated directly by Prx without the need for involvement of further intermediate proteins and Prx2 oxidation was also shown to lead to oxidation of the cytoskeletal-regulatory protein CRMP2, but the authors could not attribute this to a redox relay [52].

It has become increasingly clear in recent years that intracellular ROS homeostasis is disrupted in muscle from old subjects and this coincides with a loss of exercise-induced signalling events such as transcription factor activation [43,53,54]. In the current study no differences in the contents of Prx1-5 were seen between muscles from old compared with adult mice using western blotting approaches (Fig. 4) although because different antibodies were used, comparisons of abundances between different isoforms cannot be made. A decrease in the levels of oxidation of Prx2 was seen in response to contractile activity in muscles from old mice compared with adult mice. This decrease in Prx2 oxidation in muscle from old mice is consistent with the partial disruption of cell signalling and transcription factor activation [53] and supports the hypothesis that Prx activation may be an early step in exercise-induced signalling. Surprisingly the level of oxidation of cytosolic Prx2 in quiescent fibers were significantly decreased in muscle from old mice compared with those from adult mice at rest whilst baseline levels of the other cytosolic isoform (Prx1) showed a tendency to be reduced and mitochondrial Prx3 remained unchanged. These data appear somewhat paradoxical given that ROS levels have been reported to be elevated in muscles from old mice implying a more oxidising environment occurs within the fibers of old mice [43,[54], [55], [56]]. One theory which may explain this apparent paradox is that of “molecular habituation” whereby a constitutive signal leads to the reduction in the responsiveness of downstream signalling by prolonging the activation of negative regulators [57]. We speculate that Trx1 may play such a role in this situation since our previous data have shown that the contents of both the cytosolic and mitochondrial forms of Trx are elevated in muscle tissue from old mice compared with those from younger mice [33].

A critical step in the preparation of samples for Prx immunoblotting is the rapid alkylation of reduced cysteines prior to cell lysis to prevent further Prx oxidation during sample preparation [58]. We investigated the efficacy of NEM, IAA and MMTS at preventing Prx oxidation during muscle sample preparation [[58], [59], [60]] and MMTS proved to be the most effective in untreated muscle cells and tissue (Supplementary Figs. 1 and 2).

Overall the data presented support the hypothesis that Prx are directly oxidised in response to contractile activity in skeletal muscle fibers and that this may lead to activation of their downstream targets. The lack of responsiveness of Prx2 to the H_2_O_2_ generated during muscle contractions in the muscle from old mice also suggests that Prx are part of the redox-regulated pathways that are disrupted in aging and hence they may play a key role in the initiation of contraction-induced signalling such as transcription factor activation. Whilst some work has been undertaken towards identifying key binding partners of the different Prx isoforms in non-muscle cells, studies are required to characterise and elucidate the pathways potentially lying downstream of Prx oxidation in contracting skeletal muscle.

## Figures and Tables

**figs1:**
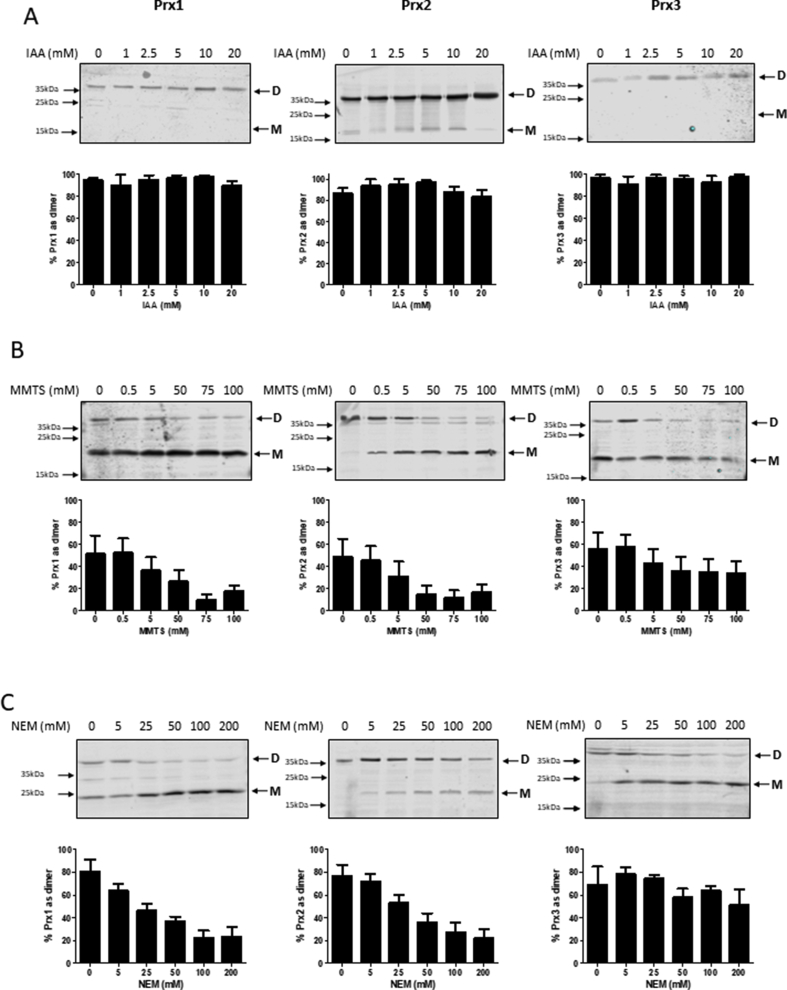
Comparison of different alkylating agents. C2C12 myotubes were incubated in the indicated concentrations of A) IAA, B) MMTS, or C) NEM and then lysed in the presence of alkylating agent before being separated by non-reducing SDS-PAGE and immunoblotted with the indicated antibodies. Densitometry analysis was carried out using a minimum of 3 replicates. Results in the bar graphs are presented as the percentage of the total Prx signal found in the dimerised form. Graphs show mean ± SEM.

**figs2:**
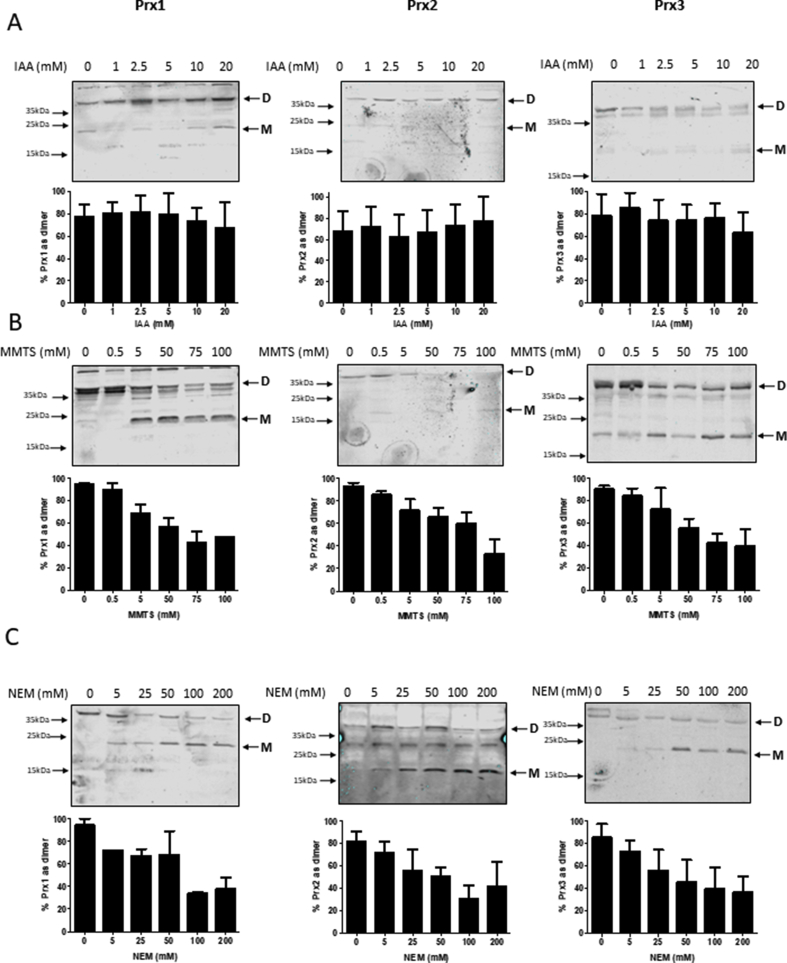
Comparison of different alkylating agents. Slices of mouse gastrocnemius muscle from adult (6-8 months) WT mice were incubated in the indicated concentrations of A) IAA, B) MMTS, or C) NEM and then lysed in the presence of alkylating agent before being separated by non-reducing SDS-PAGE and immunoblotted with the indicated antibodies. Densitometry analysis was carried out using a minimum of 3 replicates. Results in the bar graphs are presented as the percentage of the total Prx signal found in the dimerised form. Graphs show mean ± SEM.
